# An R package for Survival-based Gene Set Enrichment Analysis

**DOI:** 10.21203/rs.3.rs-3367968/v1

**Published:** 2023-09-26

**Authors:** Xiaoxu Deng, Jeffrey A. Thompson

**Affiliations:** University of Kansas Medical Center; University of Kansas Medical Center

## Abstract

Functional enrichment analysis is usually used to assess the effects of experimental differences. However, researchers sometimes want to understand the relationship between transcriptomic variation and health outcomes like survival. Therefore, we suggest the use of Survival-based Gene Set Enrichment Analysis (SGSEA) to help determine biological functions associated with a disease’s survival. We developed an R package and corresponding Shiny App called SGSEA for this analysis and presented a study of kidney renal clear cell carcinoma (KIRC) to demonstrate the approach. In Gene Set Enrichment Analysis (GSEA), the log-fold change in expression between treatments is used to rank genes, to determine if a biological function has a non-random distribution of altered gene expression. SGSEA is a variation of GSEA using the hazard ratio instead of a log fold change. Our study shows that pathways enriched with genes whose increased transcription is associated with mortality (NES > 0, adjusted p-value < 0.15) have previously been linked to KIRC survival, helping to demonstrate the value of this approach. This approach allows researchers to quickly identify disease variant pathways for further research and provides supplementary information to standard GSEA, all within a single R package or through using the convenient app.

## 1 Introduction

Functional enrichment analysis of gene expression data is a standard step following most transcriptome profiling experiments in biomedical research. It enables researchers to characterize the biological functions affected by the experiment based on a subset of relevant genes and study the drug and disease mechanisms at the molecular level ^1^. Gene sets Over-representation Analysis (ORA) and Gene Set Enrichment Analysis (GSEA) are two widely used tools to perform functional enrichment analysis ^2–4^. ORA tests whether a pre-selected gene set is over-represented in a list of differentially expressed genes than what would be expected by chance based on one or more statistical tests, such as the hypergeometric test ^5^. However, the significance threshold or certain criteria for defining a set of differentially expressed genes is arbitrary and may miss important information for genes at the boundary ^6^. To address this issue, GSEA has been proposed ^7^. By analyzing the enrichment of a gene set annotation at the top or bottom of a ranked list of genes, it finds pathways that have coordinated change in genes across any two phenotypes ^8^. Both methodologies rely on gene annotation databases, in which a great deal of work has been devoted to organizing genes and proteins into biological functions and pathways. Examples include Reactome, the Kyoto Encyclopedia of Genes and Genomes (KEGG), the Gene Ontology (GO) and the Molecular Signatures Database (MSigDB). KEGG was developed in 1995 ^9^, and GO ^10^ was founded in 1998, between which year the first eukaryotic genomes were released to the public. Reactome ^11^ was created in 2004, while MSigDB ^7^ was introduced in 2005 as a resource for GSEA. Each of these gene annotation databases has certain advantages for particular studies, however, any of them can be used to perform functional enrichment analysis.

Although functional enrichment analysis is typically focused on determining the impact of experimental differences, sometimes researchers are interested in how transcriptomic variation is associated with health outcomes, such as survival ^12,13^. Additionally, there is often an interest in which biological functions are associated with the outcome, rather than individual gene ^14^. Even if a specific gene is the cause of poor disease outcomes, an intervention might be possible at multiple points in the functional pathway involving that specific gene. Alternatively, there may be small cumulative impacts on a biological pathway that result in poor outcomes. However, typical functional enrichment analyses are focused on identifying biological functions associated with experimental differences, not outcomes. Although these ideas can align, the focus on disease differences can lead to an analysis missing important results. For example, in the case of cancer, some of the pathways that differ between tumor and normal samples will likely be associated with survival ^15^. However, other pathways that influence survival differences among survivors may not differ much between tumor and normal samples. That is because these differences relate to population variation that might be key to survival and these differences are not driven by the disease ^16^. Furthermore, the analysis will not directly identify the association between pathways and survival outcomes, or its magnitude, and researchers often resort to tests of individual genes in some of their top pathways or other indirect methods. For example, several studies used GSEA to identify cancer-related pathways and then followed by survival analysis, but their methods do not directly incorporate survival information during pathway enrichment analysis, and hence their results do not reflect the degree of association between survival and biological functions^17,18^. Furthermore, the survival differences among those with the disease are not what drove the results of the GSEA analysis or survival analysis tests only among the treatment differences rather than case-only, which creates a blind spot in the results.

There have been a couple of attempts to directly link functional enrichment to clinical outcomes (e.g. survival status or survival time). For example, Woltmann, *et al*. ^19^ identified enriched pathways through different pathway enrichment analysis tools based on a genome-wide association study (GWAS) of short-term vs. long-term breast cancer survival in order to make a conclusion to a more general breast cancer population globally. However, their studies mainly focused on verifying breast cancer-related pathways by showing the similarities between the GWAS method and different enrichment analyses and did not suggest an approach to handle censored survival data. Furthermore, though Goeman *et al*. ^20^ developed a statistical test of the association between groups of genes and a clinical outcome based on the Cox proportional hazards model, their proposed method requires a set of pre-selected genes and does not consider coordinated changes of the genes in a pathway.

We suggest there is a simple solution to this challenge. Researchers can perform Survival-based Gene Set Enrichment Analysis (SGSEA) to understand how transcriptomic variation among patients can be used to identify biological functions associated with survival from the disease. Using this approach, researchers may discover unexpected patterns of gene activity, leading to new avenues of research. In this paper, we demonstrate how while GSEA can often provide insight into disease etiology, SGSEA can provide unique insights into disease outcomes. Although this method is already readily available to researchers, we believe one reason it is not widely used is that it is not included as a standard tool in most software and there has not been a paper to describe it. Therefore, we present the SGSEA R package and Shiny app that can be used to conveniently conduct these analyses.

## 2 Methods

### 2.1 Survival Gene Set Enrichment Analysis

GSEA is a powerful tool to identify the biological functions that are enriched in up- or down-regulated gene expression from comparing two treatment groups of genes. Typically, the log-fold change in expression between treatments is used to rank genes. Then GSEA essentially tests whether the distribution of ranks (of log fold changes) in a pathway is different than one would expect by chance. Because the test is based on ranks, any statistics can be used in place of log fold change. Therefore, to perform the Survival-based Gene Set Enrichment Analysis (SGSEA), we used the log hazard ratios to find the biological functions that are associated with mortality or survival. The advantage of the Cox model is that no assumptions are required on the distribution of hazards and we can assess the effect of risk factors by assuming the shape of the hazard does not change over time. Also, it can handle censored data. Through this semi-parametric model, we can evaluate the change in the instantaneous risk of mortality while incorporating each individual gene’s expression as the risk factor. The hazard of dying for gene i at time t is defined as

1 $$h\left(t,x_{i}\right)=h_{0}(t)e^{\beta x_{i}}$$

where $h_{0}(t)$ is the baseline hazard function when ${gene}_{i}$ is not expressed.

Taking the logarithm of Eq. (1) on both sides, we have

2 $$log[h(t,x_{i})]=log[h_{0}(t)]+\beta x_{i}$$

β in Eq. (2) is the log hazard ratio for a one-unit increase in the value of $x_{i}$, the normalized gene expression. In summary, the SGSEA is a variation of GSEA by performing GSEA with a hazard ratio instead of a log fold change.

### 2.2 R package

All analyses performed in this paper use R statistical programming language ^21^. We created the *SGSEA* R package that is designed specifically for Survival-based Gene Set Enrichment analysis. It provides a complete set of functions for SGSEA and standard GSEA that allow users to easily normalize gene expression data using a mean-variance model method, calculate log hazard ratios based on the Cox proportional hazard model, calculate log_2_ fold-change based on the negative binomial regression model, extract reference pathway annotations from the Reactome database, generate the pathway analysis statistics, and produce a table of both the top 10 significant pathways associated with disease survival and top 10 significant pathways associated with disease mortality as well as the individual pathway enrichment plot. Therefore, this package can also be used for the standard case vs. control (or normal vs. tumor) GSEA method, providing users with the additional advantage of conducting various functional enrichment analyses using a single package.

The **coxph** function from the *survival* package is used to fit Cox Proportional Hazards model ^22^, from which estimates of the log-hazard are obtained. Because higher hazard ratios generated by the model indicate that increased expression is associated with an increased hazard of the event, higher enrichment scores indicate that increased expression in a pathway is associated with a higher risk of mortality. We then call the **fgsea** function from the *fgsea* package in R to conduct the enrichment analysis. This package can efficiently and accurately estimate p-values, which significantly increases the sensitivity of GSEA in multiple hypothesis correction procedures compared to other implementations^23^. In addition to our case-only SGSEA, we also conducted the standard GSEA method between the tumor and tumor-adjacent normal tissues by using the **DESeq** function from the *DESeq2* package in R ^24^, which uses a negative binomial generalized linear model, to get the log fold-change as the input of the enrichment analysis. This package can be downloaded at the GitHub repository https://github.com/ShellsheDeng/SGSEA.git.

### 2.3 Shiny App

**Figure 1:**
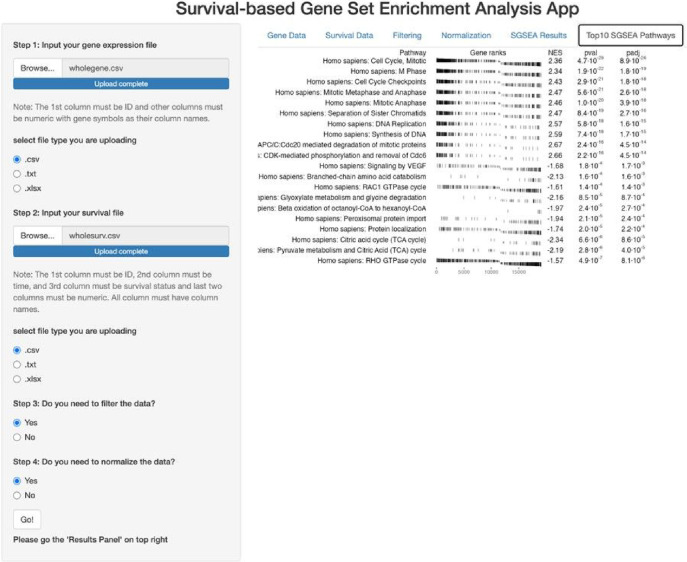
SGSEA Shiny App Interface

The SGSEA shiny app is an interactive web application that performs the Survival-based Gene Set Enrichment Analysis (Fig. 1). It was built using the *Shiny* package and allows users to input multiple formats of the gene expression file and corresponding survival information. The SGSEA shiny app outputs a table of enrichment results with a series of options for data pre-processing steps such as filtering and normalization. The app provides a visually appealing and intuitive interface for users to interact with on the left and show results on the right. It is designed for researchers with no programming background. This app can be initiated by either calling the **runExample** function from the *SGSEA* package or by visiting the website https://optik.shinyapps.io/SGSEA/.

### 2.4 Data Summary

To demonstrate the *SGSEA* R package and app, we used an mRNA-seq dataset that contains patients with Kidney Renal Clear Cell Carcinoma (KIRC) (version 2016_01_28) from The Cancer Genome Atlas (TCGA) ^25^. Data were obtained from The Broad Institute’s Genome Data Analysis Center (GDAC) Firehose website (https://gdac.broadinstitute.org/). In this dataset, there are 520 observations, out of which 360 patients were alive at the last follow-up and 160 patients were dead. After filtering out some invalid data input (e.g., survival time < 0) and genes without HUGO symbols, there were 20,502 genes remaining from the original 60,660. The standard GSEA method conducted in this paper uses 70 out of those 520 patients who have paired normal tissues that allow us to conduct a case-control analysis to derive log fold-change of differential expression between tumor and normal.

We used the human pathway annotations from the Reactome pathway database. We mapped Entrez IDs to HUGO gene symbols using the org.HS.eg.db R package^26^. Reactome is a curated and peer-reviewed pathway database that provides detailed information on molecular processes, such as metabolism, signaling, and disease pathways for over 2,000 different species ^27^. Due to the high-quality annotations and comprehensive coverage of human biological pathways, we selected Reactome as the knowledge base in this analysis to demonstrate how SGSEA can help researchers gain insights into underlying biological processes and mechanisms in their data.

### 2.5 Data Structure

The general data structure for conducting the SGSEA method is the same for both the R package and the Shiny app. The rows should be the patients or samples and the columns should be the gene expression count data. To perform the survival analysis, there are two important variables that need to be included in the data, the patient’s time-to-event and survival status. Users can also check the input data structure by referring to an example data called KIRC that is embedded in the package which is a subset of the original KIRC RNA-seq data.

### 2.6 Data Preprocessing

#### 2.6.1 Filtering

The R package users can choose different filtering methods based on their own preferences. However, the filtering rule is fixed in the Shiny app and the results presented in this paper all use the same filtering rule. Specifically, we excluded genes with less than 1 read per 10 participants on average before conducting the SGSEA method. For the KIRC data set, there were 19,098 genes that withstood our filtering procedure. When performing the typical GSEA method, this filtering rule left the total number of genes at 18,198. This difference is due to the fact that the analyses were conducted on different sets of samples.

#### 2.6.2 Normalization

In order to adjust for the heteroscedasticity and nonnormality of the gene expression data, we use the **voom** function from *limma* package ^28^ for both the *SGSEA* package and the Shiny app before performing the SGSEA method. This function estimates the mean-variance trend for log counts and then assigns a weight to each observation based on its predicted variance. However, the normalization method for the preprocessing step before performing typical GSEA in the *SGSEA* package is different from the one in the preprocessing step of the SGSEA method, because it calls out the **DESeq** function from *DESeq2* package which uses raw counts and models the normalization inside the negative binomial model.

## 3 Results

### 3.1 Survival-based Gene Set Enrichment Analysis

**Figure 2:**
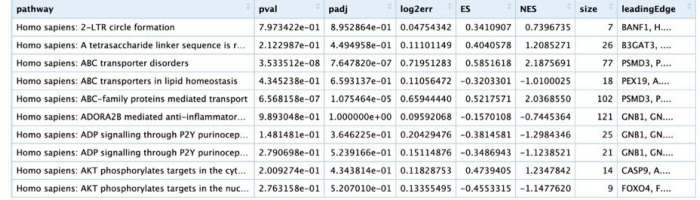
*SGSEA* package and Shiny app pathway analysis statistics using KIRC data

**Figure 3:**
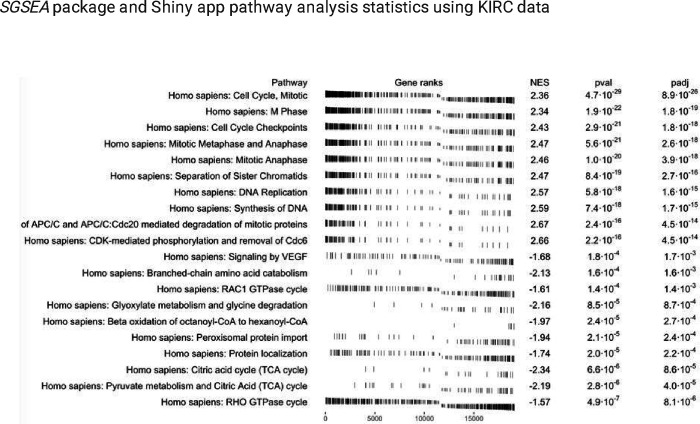
*SGSEA* package and Shiny app pathway analysis output using KIRC data: the top 10 significant pathways with positive NES and the top 10 significant pathways with negative NES

As an example of the *SGSEA* package and Shiny app, the results of the analysis using the KIRC dataset are presented as follows. An example output of the enrichment scores table for the 10 possible pathways can be found in Fig. 2. The top 10 significantly enriched pathways with positive Normalized Enrichment Score (NES) and the top 10 with negative NES are shown in Fig. 3. The density of lines in Fig. 3 implies the direction of the enrichment in the pathway. For example, the density of the lines for the top 10 significant pathways with positive NES is comparatively higher on the left than those on the right, which reflects the fact that these top 10 pathways are highly enriched with genes with larger log hazard ratios. Likewise, the density for the top 10 significant pathways with negative NES is comparatively higher on the right than those on the left, indicating that these top 10 pathways are highly enriched with genes with smaller log hazard ratios. A larger log hazard ratio for a certain gene means that KIRC patients who have an increasing expression of this gene tend to have a higher risk of dying. Similarly, a smaller log hazard ratio for a gene means that those who have an increasing expression of this gene tend to have a higher chance of survival. Therefore, the top 10 significant pathways with positive NES are the ones most associated with mortality for people with increased expression in those genes while the top 10 significant pathways with negative NES are the ones most associated with survival for people with increased expression in those genes. Figure 3 also indicates that proliferation-related biological functions are associated with KIRC mortality, while metabolism and oncogenic transformation pathways are associated with KIRC survival, for people who have increased expression.

**Figure 4:**
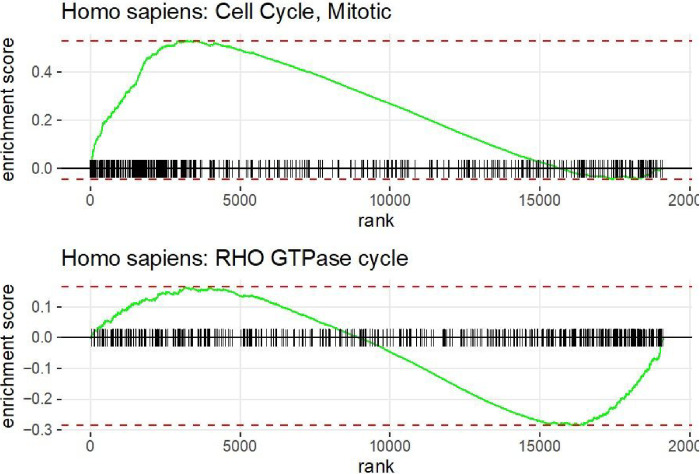
*SGSEA* package pathway analysis output using KIRC data: The individual enrichment plot of the top 1 significant pathway with positive NES (top) and negative NES (bottom)

Enrichment plots for top significant pathways with a positive NES (on the top) and a negative NES (on the bottom) are shown in Fig. 4. The height from the peak of the plot to the horizontal line is the enrichment score for a specific pathway. The plot on the top is the Mitotic Cell Cycle pathway which is related to cellular proliferation, showing that it is significantly enriched in kidney cancer genes with the largest log hazard ratios (NES = 2.36, adjusted p-value = 8.9*e*^−26^). Conversely, the pathway on the bottom with negative NES is the RHO GTPase cycle which is significantly enriched in genes with the smallest log hazard ratios (NES=−1.57, adjusted p-value = 8.1*e*^−6^). The biological function of this pathway is related to oncogenic transformation ^11^. Outputs like Fig. 4 can be produced by the *SGSEA* R package (which calls the *fgsea* package to create these plots) to visualize the results for specific pathways of interest from SGSEA.

### 3.2 Tumor vs. Normal Gene Set Enrichment Analysis

**Figure 5:**
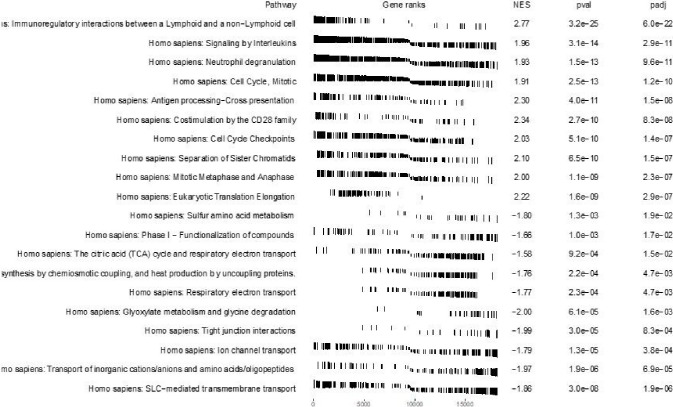
GSEA pathway analysis using *SGSEA* package on KIRC data: Top 10 significant pathways with positive NES and top 10 significant pathways with negative NES

The result of the standard GSEA method using the *SGSEA* package based on the log fold change between tumor and tumor-adjacent normal tissue is shown in Fig. 5. The signaling pathway pertinent to immunoregulatory interactions between a Lymphoid and a non-Lymphoid cell is significantly enriched with up-regulated genes in tumor compared to normal. On the contrary, cellular metabolism pathways (on the bottom) are the most significantly enriched pathways with down-regulated genes in tumor compared to normal.

### 3.3 Comparison between case-only SGSEA and tumor vs. normal GSEA

**Figure 6:**
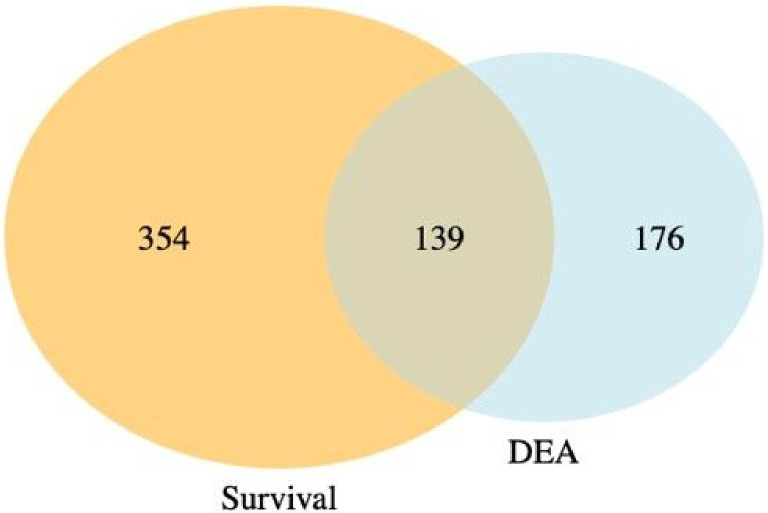
Comparison between SGSEA and GSEA

Figure 6 presents a Venn diagram showing the overlapping significant pathways identified by case-only SGSEA and tumor vs. normal GSEA separately at a 15% significance level to keep in line with the threshold recommendation from the original GSEA paper^7^. Out of the total 1867 pathways, 139 significant pathways were found to be overlapping. Additionally, SGSEA identified 354 significant pathways that were not detected by GSEA.

In Table 1, SGSEA identified the Mitotic Cell Cycle and Cell Cycle Checkpoints as two significant pathways enriched in genes with the largest log hazard ratios, while standard GSEA found these two pathways are statistically significantly (adjusted p-value < 0.15) enriched with up-regulated genes in tumor compared to normal. That is, these two pathways that are significantly associated with mortality were also found to be up-regulated in tumor vs. normal samples, suggesting that these two pathways are up-regulated with tumor vs. normal during tumor progression and will increase the risk of dying. On the other hand, SGSEA identified the RHO GTPase cycle and Branched-chain amino acid catabolism as two pathways significantly (adjusted p-value < 0.15) associated with survival. However, typical GSEA found that the former one is not statistically significantly (adjusted p-value > 0.15) enriched in up-regulated genes with an NES score of 1.09, while the latter is significantly (adjusted p-value < 0.15) enriched in down-regulated genes with an NES score of −1.67, showing that the two methods can provide different insights.

## 4 Discussion

In this work, we have presented the *SGSEA* R package, the Shiny app, and how they work using a kidney cancer dataset as an example. Our Shiny app provides a user-friendly interface to conduct SGSEA efficiently while our R package is more flexible for R programmers to conduct and compare both SGSEA and GSEA. With the *SGSEA* package, researchers and analysts can quickly and easily perform SGSEA as well as standard GSEA within one package and gain cross-reference information. We also identified significant pathways related to kidney cancer progression and survival and demonstrated how the SGSEA method provides complementary information to a typical GSEA. In summary, pathways significantly associated with mortality (NES > 0 and adjusted p-value < 0.15) are enriched in genes whose expression is linked with higher mortality rates in KIRC patients. Similarly, pathways significantly associated with survival (NES < 0 and adjusted p-value < 0.15) are enriched in genes whose expression is linked with higher survival rates in KIRC patients. That is, the risk of dying will increase for KIRC patients who have increasing gene expression in pathways that are statistically significantly associated with mortality or decreasing gene expressions in pathways that are significantly associated with survival. Specifically, the pathways most closely linked to mortality are related to proliferation. This result has been validated in previous studies demonstrating that proliferation is associated with mortality in KIRC ^29–32^.

A key difference between SGSEA and a typical GSEA is that SGSEA is case-only and GSEA is a comparison between treatment/case and control. However, even when both types of data are available, as they are in these data, SGSEA identifies pathways associated with a disease’s survival which might not be enriched using typical GSEA, such as pathway RHO GTPase cycle ^33^. That is because the drivers of survival differences do not necessarily need to be the processes that are changed by disease. Therefore, our finding suggests the potential utility of SGSEA in identifying key pathways involved in cancer progression and survival. For example, when comparing the significant pathways generated from the case-only SGSEA and those generated from tumor vs. normal GSEA, there are 139 (Fig. 6) overlapping pathways that are both related to disease outcome and disease state, conveying important biological information that might help researchers start their research more efficiently by focusing on the cell products or changes generated by these biological pathways first instead of those 176 (Fig. 6) significant pathways identified by typical GSEA. However, not all pathways associated with mortality are found to be up-regulated in tumor vs. normal tissues, nor are all pathways associated with survival found to be down-regulated in tumor vs. normal samples. Those 354 (Fig. 6) significant pathways from the Venn diagram, which do not differ between tumor and normal tissue but differ between survival and mortality, provide complementary information that helps researchers explore the biological processes underlying disease survival.

However, it is important to note that there are certain constraints associated with this approach. These limitations primarily stem from the dependence on pre-existing pathway databases and the utilization of the GSEA method, which assesses coordinated change in gene expression. Also, currently only the Reactome database is accessible for the *SGSEA* package. The aim of this work is to demonstrate the utility of performing SGSEA and provide tools to facilitate such analyses. SGSEA helps researchers examine the association between a disease’s survival and particular biological functions. Although Cox proportional hazards models can be used to identify individual genes associated with mortality, by studying the biological functions most associated with mortality and survival, researchers may more accurately be able to determine the key drivers of the processes involved, which will not always contain the genes with the highest hazard ratios. In summary, our study confirms that pathways associated with a disease outcome are not always associated with a disease state, and our *SGSEA* package and the Shiny app can help researchers conduct such functional enrichment analyses.

## Figures and Tables

**Table 1:** An example result of pathways tested by SGSEA and GSEA

	SGSEA		GSEA	
	Adjusted P-value	NES	Adjusted P-value	NES
**Homo sapiens: Cell Cycle, Mitotic**	8.9 × 10^−26^*	2.36	1.2 × 10^−10^*	1.91
**Homo sapiens: Cell Cycle Checkpoints**	1.8 × 10^−18^*	2.43	1.4 × 10^−7^*	2.03
**Homo sapiens: RHO GTPase cycle**	8.1 × 10^−6^*	−1.57	0.4×10^0^	1.09
**Homo sapiens: Branched-chain amino acid catabolism**	1.6 × 10^−3^*	−2.13	0.9 × 10^−1^*	−1.67

* significant at 0.15

## Data Availability

The data that support the findings of this study is Kidney Renal Clear Cell Carcinoma dataset (KIRC, version 2016_01_28), which is part of The Cancer Genome Atlas (TCGA) project and is openly available at The Broad Institute’s Genome Data Analysis Center (GDAC) Firehose website (https://gdac.broadinstitute.org/).
